# Age differences in anticipatory and executory mechanisms of gait initiation following unexpected balance perturbations

**DOI:** 10.1007/s00421-020-04531-1

**Published:** 2020-10-26

**Authors:** Luca Laudani, Lorenzo Rum, Maria Stella Valle, Andrea Macaluso, Giuseppe Vannozzi, Antonino Casabona

**Affiliations:** 1 Cardiff School of Sport and Health Sciences, Cardiff Metropolitan University, Cardiff, UK; 2 Department of Movement, Human and Health Sciences, University of Rome “Foro Italico”, Rome, Italy; 3 Department of Biomedical and Biotechnological Sciences, Section of Physiology, University of Catania, Catania, Italy

**Keywords:** Posture, Fall risk, Locomotion, Elderly, Electromyography, Spatiotemporal analysis

## Abstract

**Purpose:**

An age-related decline in anticipatory postural mechanisms has been reported during gait initiation; however, it is unclear whether such decline may jeopardize whole-body stability following unexpected balance perturbations. This study aimed to compare young and older individuals’ ability to generate postural responses and preserve stability in response to external waist perturbations delivered within gait initiation.

**Methods:**

Ten young and ten older participants performed 10 gait initiation trials followed by 48 unperturbed and 12 perturbed trials in a random order. A stereophotogrammetric system and three force platforms were used to quantify mechanical parameters from the preparatory phase (e.g., timing and amplitude of postural adjustments) and from the stepping phase (e.g., step characteristics and dynamic stability). Activation patterns of lower leg muscles were determined by surface electromyography.

**Results:**

Older participants responded to perturbation with lower increase in both magnitude (*p* < 0.001; η^2^_p_ = 0.62) and duration (*p* = 0.001; η^2^_p_ = 0.39) of preparatory parameters and soleus muscle activity (*p* < 0.001; η^2^_p_ = 0.55), causing shorter (*p* < 0.001; η^2^_p_ = 0.59) and lower (*p* < 0.001; η^2^_p_ = 0.43) stepping, compared to young participants. Interestingly, young participants showed greater correlations between preparatory phase parameters and dynamic stability of the first step than older participants (average *r* of − 0.40 and − 0.06, respectively).

**Conclusion:**

The results suggest that young participants took more time than older to adjust the anticipatory biomechanical response to perturbation attempting to preserve balance during stepping. In contrast, older adults were unable to modify their anticipatory adjustments in response to perturbation and mainly relied on compensatory mechanisms attempting to preserve stability via a more cautious stepping strategy.

## Introduction

The initiation of human gait from stance begins with an anticipatory postural adjustment (APA) involving a preparatory weight shift and forward lean, i.e. a “controlled fall”, for a balanced first-step execution (Mann et al. 1979; Winter 1995; Laudani et al. 2006). To initiate gait from quiet stance, a stereotyped activity of the leg muscles produces an anticipatory shift of the centre of pressure (COP) backward, which propels the body centre of mass (COM) forward (Brenière et al. 1987; Crenna and Frigo 1991). At the same time, hip abductor muscles also create mediolateral ground reaction force that determines an anticipatory COP displacement towards the stepping leg and an opposite COM motion towards the stance leg, minimising the potential imbalance at the instant of single-limb stance (Jian et al. 1993; John et al. 2012; Maslivec et al. 2018). Referred to as lateral thrust, this preparatory/anticipatory weight shift sideways is ballistically controlled based on the lateral force to overcome (Mouchnino et al. 1992; Lyon and Day 1997, 2005) and responds differently to either externally or internally generated perturbation stimuli. For instance, it has been shown that the lateral thrust is resistant to sudden variations in sensory information, such as proprioceptive-afferent inflow evoked by muscle vibration (Ruget et al. 2008) or galvanic stimulation (Bent et al. 2004). On the other hand, Mouchnino et al. (2012) showed that young adults were able to actively increase their thrust amplitude (e.g., ground pressure and muscle activity) when an external lateral waist pull was unexpectedly delivered within the preparatory phase of gait initiation. Similarly, when an unpredictable waist perturbation was delivered at the thrust onset, the first-step initiation was delayed in young adults (i.e., increased duration of the preparatory phase) likely to allow the overall postural adjustment to achieve an anticipated state condition reflecting an estimation of whole-body stability necessary to release a balanced step (Mille et al. 2014). Therefore, the results from these previous studies indicated that in real-life circumstances, young adults are able to modify both lateral thrust amplitude and duration to reduce their risk of falling when the body is subjected to an external perturbation that would effectively challenge body balance.

The control of balance is particularly important in older individuals as they are more likely to fall while walking short distances including locomotor transitions, such as gait initiation (Tinetti et al. 1995). Despite these falls frequently occur while moving the body in the sagittal plane, the most common cause of falling is incorrect transfer or shifting of body weight (Robinovitch et al. 2013), suggesting that quality of the anticipatory weight shift prior to stepping plays a key role in determining the risk of falling during real-life conditions. Previous studies’ reports on older individuals have revealed a characteristic age-related deterioration of the anticipatory/preparatory mechanisms of gait initiation. For instance, a tendency for the lower limbs muscle activity to be more variable during the preparatory phase than during the first-step execution in older people with respect to young people has been reported by previous investigators (Polcyn et al. 1998; Mickelborough et al. 2004). Laudani et al. (2006) demonstrated impaired upper body coordination patterns employed by older adults during the preparatory phase of gait initiation, leading to decreased head stability and challenging whole-body balance to a greater extent compared to younger adults. These results were confirmed by Maslivec et al. (2018) who also showed impaired/delayed anticipatory activation of the neck muscles during the preparatory phase of gait initiation in older individuals. Despite these previous studies have highlighted an age-related decline in the anticipatory mechanisms during self-paced gait initiation, it is still unknown whether such decline could jeopardize whole-body stability under real-life conditions during gait initiation, e.g., in occurrence of real body perturbations potentially leading to imbalance and fall.

In the present study, we dealt with this issue by comparing the ability of young and older individuals to generate the appropriate APAs aimed at maintaining whole-body stability in response to unexpected waist pull perturbation occurring within the preparatory phase of gait initiation. Based on previous studies’ findings (Mouchnino et al. 2012; Mille et al. 2014), it was hypothesised that young participants would be able to modify both timing and amplitude of their anticipatory response to the perturbation in relation to an adequate maintenance of whole-body stability during the first step. In contrast, we hypothesised that older adults would show decreased ability to actively respond to the perturbation in terms of ground reaction force production and magnitude of lower limb muscle activity, thus compromising the first-step execution and whole-body stability.

## Methods

### Participants

Ten healthy young (age: 25 ± 2 years, mass: 60.2 ± 7.4 kg, height: 1.65 ± 0.08 m, gender: 8 females, 2 males) and ten healthy community-dwelling older adults (age: 73 ± 5 years, mass: 68.3 ± 12.5 kg, height: 1.63 ± 0.11 m, gender: 8 females, 2 males) volunteered to participate in the study. The sample size was determined by a priori statistical power analysis for a two-factor mixed ANOVA (G*Power software version 3.1.9.4; α = 0.05, power = 0.80, effect size = 0.50, correlation among repeated measures = 0.40). Older participants were selected according to the inclusion/exclusion criteria to define ‘‘medically stable’’ older individuals for exercise studies, as proposed by Greig et al. (1994). Participants were included in the study only if they had no history of neurological and/or orthopaedic disorders precluding their balance capability during standing and walking tasks, as evaluated by health status questionnaire. In addition, the International Physical Activity Questionnaire was filled by all participants to select only individuals who were not engaged in regular training or sport practice more than 3 times a week, for more than 40–60 min each time (Laudani et al. 2013). The Berg Balance Scale was used to assess gross balance (static and dynamic) during a set of daily life movement tasks (Berg et al. 1989), and all participants were classified as low fall risk, i.e. with a score of 41–56. Each volunteer gave written informed consent and the local Ethics Committee of Cardiff Metropolitan University approved all experimental procedures in agreement with the standards set by the Declaration of Helsinki.

### Experimental protocol

Participants were required to initiate gait from a standing position and to take no less than four steps forward at their most comfortable speed while focusing on a visual target set at the eye level and located three meters away from the starting stance position. Before stepping forward, participants were asked to stand as still as possible with their trunk vertical, their arms naturally hanging of the side and their feet normally apart. Each participant was asked to initiate walking with their dominant leg, which was determined by asking which leg they would use to shoot the ball (Melick et al. 2017).

**Fig. 1:**
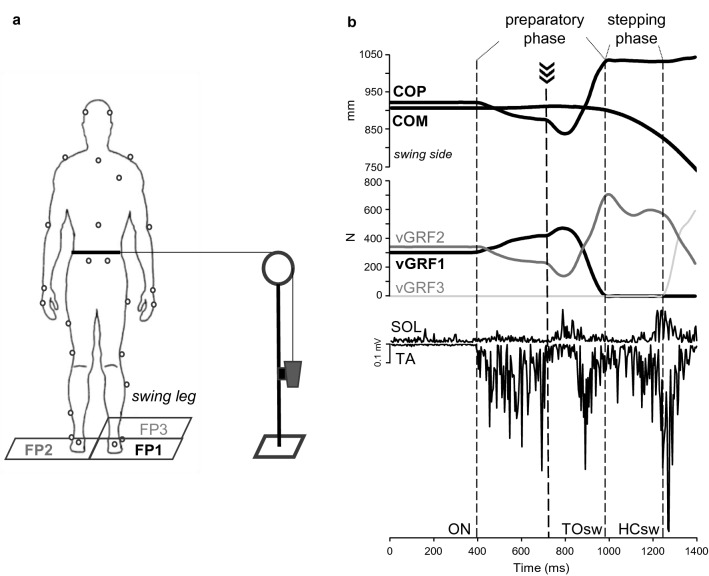
Experimental setup (**a**) and measurements (**b**). Gait initiation was performed from a quiet standing posture. During preparation of the first step, participants were pulled laterally after releasing of a load hold firm by an electromagnetic brake. The perturbation fallen within the second third of the preparatory phase (thick dashed line in **b**). Three independent force plates (FP1, FP2, FP3) allowed to measure changes in ground reaction forces (GRF) represented in **b** with only the vertical components (vGRF1, vGRF2, vGRF3). Center of pressure (COP) was determined from the GRF, and center of mass (COM) was computed from body marker coordinates. The period of preparation for the first step (preparatory phase) is delimited by two dotted lines, indicating the onset of weight transfer to the swing leg (ON) and the toe-off of the swing leg (TOsw). The period of swing leg (stepping phase) is included between the TOsw and the heel contact of the swing leg (HCsw). The rectified raw EMG activity of tibialis anterior (TA) and soleus (SOL) muscles was recorded over the two phases and used for further elaboration

A safety harness was fitted to the participant’s trunk and attached to a ceiling-mounted trolley for preventing any fall occurrence while not constraining the movement. As shown in Fig. 1a, a belt attached to the participants’ waist was connected by an inextensible rope passing through a pulley to a load of 10% relative to the participant body mass that was held firm by an electromagnetic brake. The load was released by switching off the brake when the vertical ground reaction force under the stepping foot increased by 10% of the participant’s body weight. This caused the participants’ pelvis to be pulled laterally towards the swing leg, i.e. in opposite direction to the COM displacement towards the stance leg that typically occurs during this phase (Mouchnino et al. 2012). The perturbation was delivered with an electromechanical delay of ~ 120 ms from the instant of load release and, hence, fell within the second third of the preparatory phase (Fig. 1b). Another electromagnet connected to the rope was triggered when vertical ground reaction force under the stepping foot dropped to zero (i.e. at toe-off of the swing leg) and detached the load from the rope, thus ending the perturbation. Very low frictional resistance materials were used, and the components were carefully secured and aligned in the frontal plane to guarantee a smooth load motion and a discrete waist pulling.

Participants were required to perform 10 consecutive self-paced gait initiation trials without any perturbation during a so-called “blocked” (BLK) condition, followed by 60 gait initiation trials with a 20% rate of perturbation occurrence that included 48 unperturbed (UP condition) and 12 perturbed trials (PT condition). Perturbation occurrence was randomized to minimise the ability to predict the perturbed trials by the participant. Successive trials were separated by a minimum of 60 s, and participants were allowed to rest 5 min every 20 trials to avoid fatigue. In the unperturbed trials, the load was detached by the rope that was hence free to move smoothly through the pulley when the participant commenced to walk forward from a standing position. The very low frictional resistance materials between the rope and the pulley, hence, allowed the participant to move and initiate locomotion without perceiving any external load throughout all phases of gait initiation in all unperturbed trials. All trials from the BLK and PT conditions and ten randomly-selected trials from the UP condition were used for further analysis.

### Experimental setup

Participants stood onto two force platforms (Kistler 9287, Kistler, Switzerland) that were positioned at ground level underneath each foot before initiating to walk; another platform (Kistler 9287, Kistler, Switzerland) was placed in front of the stepping limb, i.e. the right limb in all participants (Fig. 1a). Ground reaction force (GRF) from all platforms was acquired with a sampling rate of 1000 Hz using a motion analysis software (VICON Nexus 2.7.1, Oxford Metrics, London, UK) that allowed calculation of the combined COP from the two feet. A 13-camera motion analysis system (MX System, Vicon Motion Systems, Los Angeles, CA) was used to collect position data of 35 markers placed on selected body landmarks according to the full body Plug-in-Gait model, with a sampling rate of 250 Hz. The participant’s COM was obtained using a 12-segment biomechanical model (Gutierrez et al. 2003). A portable electromyography (EMG) device (Trigno Wireless System, Delsys, Boston, MA) was used to collect electric signals from the tibialis anterior (TA) and the soleus (SOL) muscles in the swing limb of participants, with a sampling frequency of 1000 Hz. EMG sensors did apply a standard pre-amplification of × 300 which was then further amplified digitally with an overall effective gain of × 909. To increase the subject’s skin impendence and to reduce motion artefacts, the experimenter gently shaved and abraded the skin before placing electrodes that were then fixed over the muscle bellies using medical tape. All data were collected and time-synchronised by the same motion analysis software (VICON Nexus 2.7.1, Oxford Metrics, London, UK).

### Data processing and analysis

GRF components from all platforms were filtered using a low-pass 4th order Butterworth filter with a 15 Hz cut-off frequency. As shown in Fig. 1b, the onset of gait initiation (ON) was evaluated as the beginning of weight transfer towards the swing leg, which was identified as when the vertical GRF of the stepping foot exceeded by one standard deviation the mean baseline value obtained over a 250 ms window prior to ON during quiet standing (Patla et al. 1993). Instant of toe-off of the swing leg (TOsw) was identified as when the vertical GRF of the same leg dropped to 0 N, indicating that the foot had lost contact with the platform. Instant of heel contact of the swing leg (HCsw) was identified as when the vertical GRF from the platform in front of the participant exceeded 0 N, indicating that the foot had touched the plate.

Gait initiation was divided into a preparatory phase, which lasted from the ON until TOsw, followed by a stepping phase, which lasted from TOsw until HCsw (Fig. 1b). During the preparatory phase, the following measures were obtained: greatest lateral displacement of COP (thrust amplitude) and time interval of this displacement (thrust duration), peak GRF components (vertical, anteroposterior and mediolateral) under the swing leg normalised by body weight (BW); total phase duration; in addition, ankle plantar flexion and dorsiflexion peak moments were obtained using the above mentioned Plug-in-Gait protocol. During the stepping phase, the following measures were obtained: step length and width from the anteroposterior (AP) and mediolateral (ML) displacement of the swing leg heel marker at heel contact, respectively; step height as the highest vertical displacement of the heel marker; AP and ML COM position at HCsw; total phase duration; step velocity as the ratio between the step length and the stepping phase duration. The margin of stability (MOS) was used to quantify dynamic stability in the AP and ML direction at HCsw adapting the method introduced by Hof et al. (2005). In particular, MOS was measured as the difference between the base of support boundary derived by the position of the swing heel marker at HCsw, and the position of the extrapolated COM (exCOM) calculated as follows:

$${\text{exCOM}} = {\text{xCOM}} + ~\frac{{{\text{x}}^{\prime}{\text{COM}}}}{{\sqrt {\frac{g}{l}} }}$$

with “xCOM” and “x′COM” representing the COM position and velocity, respectively, “*g*” being gravitational acceleration of 9.81 ms^−1^, and “*l*” corresponding to the limb length calculated according to the anthropometric tables provided by Winter (2009). AP and ML MOS corresponded to the difference between the AP and ML position of the heel marker and the AP and ML position of the exCOM at HCsw, respectively. As reported by other authors in previous studies (McCrum et al. 2014; Yiou et al. 2017), negative values of MOS indicated an unstable body configuration state whereby the participant should make additional motor actions to preserve stability and avoid falling, e.g., initiate the step forward. Therefore, in our study, the more negative the MOS values were, the further the exCOM was from the base of support boundary, and the lower dynamic stability was achieved at the end of the first step (and vice versa).

EMG signals were band-pass filtered using a 4th order Butterworth filter already integrated within the Trigno sensors (20–450 Hz) followed by full-wave rectification. Filtered EMG signals were time-normalised by interpolation at one percent intervals. Magnitude of muscle activation was computed for each trial as the root mean square (RMS) of the processed EMG signal over 10% consecutive windows during the preparatory phase. All RMS values from each participant were normalised to their maximum ensemble average value obtained in the BLK condition.

All data analyses were implemented using Matlab version R2019a (Mathworks Inc, Natick, MA).

### Statistical analysis

Normal distribution of data was checked using the Shapiro–Wilk test and with z-score transform of skewness and kurtosis values (all values <|2|). A two-factor mixed analysis of variance (ANOVA) was performed to evaluate the effects of age group (young and older), condition (BLK, UP and PT) and their interaction as independent variables, on MOS and COM positions at HCsw in both AP and ML directions as primary dependent variables and on mechanical parameters of preparatory (thrust duration, thrust amplitude, peak vertical force, peak anteroposterior force, peak mediolateral force, total duration, peak ankle plantar flexion and dorsiflexion moments) and stepping phase (step duration, step width, step height, step velocity) as secondary dependent variables. Differences between conditions were evaluated within each group using a repeated measures ANOVA followed by post hoc Student’s *t* test with Bonferroni correction as pairwise comparison.

To evaluate change in EMG amplitude between groups and experimental conditions for each muscle (TA and SOL), a three-way mixed ANOVA was performed with age group (young and older) as between-subject factor and experimental condition (BLK, UP and PT) and RMS windows as within-subject factors. To explore the specific contribution of condition and RMS window in differentiating the two groups, separate one-way ANOVAs were performed for each muscle with multiple comparisons applying Bonferroni corrections. Data were checked by the Mauchly's test and, if sphericity assumption was violated, the Greenhouse–Geisser adjustment was applied on repeated measures. To quantify the relationship between muscle activity and joint moments, linear regressions were performed between the average normalised EMG activity obtained in each muscle and both ankle plantar flexion and dorsiflexion moments for each group.

The relationship between preparatory and stepping phases of gait initiation within each condition (BLK, UP and P) was investigated by a partial correlation analysis after removing the variations due to subjects (Bland and Altman, 1995), with the first-step parameters (step duration, step width, step height, step velocity, AP and ML COM position at HCsw, AP and ML MOS at HCsw, total phase duration) as dependent/outcome variables and the preparatory phase parameters (thrust duration, thrust amplitude, peak vertical force, total phase duration) as independent/predictor variables.

Statistical analyses were undertaken using SPSS version 20.0 (SPSS, Inc., Chicago, IL-IBM, Somers, NY, USA). Level of significance was set at α < 0.05 and effect size (partial eta squared η^2^) was also computed.

## Results

### Parameters of stability

**Fig. 2:**
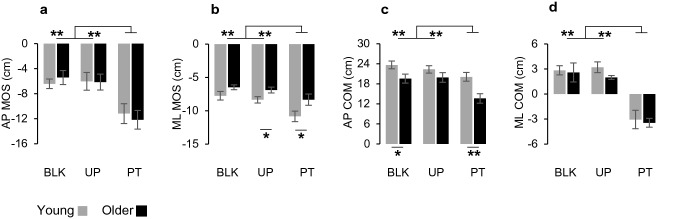
Summary of statistic results for stability parameters. Plots represent margin of stability in anteroposterior (**a**, AP MOS) and mediolateral (**b**, ML MOS) axes, center of mass displacement in anteroposterior (**c**, AP COM) and mediolateral (**d**, ML COM) axes. Comparisons between young (grey) and older (black) participants in blocked (BLK), unperturbed (UP) and perturbed (PT) conditions. Data are reported as means and standard errors. Statistically significant results are labeled as follows: *, *p* < 0.05; **, *p* < 0.01

Mixed two-way ANOVA showed a main effect of condition for all the stability parameters (Fig. 2): AP MOS (F_2,36_ = 17.636, *p* < 0.001; η^2^_p_ = 0.49; Fig. 2a), ML MOS (F_2,36_ = 20.972, *p* < 0.001; η^2^_p_ = 0.54; Fig. 2b), AP COM (F_2,36_ = 16.324, *p* < 0.001; η^2^_p_ = 0.48; Fig. 2c), ML COM (F_2,36_ = 48.715, *p* < 0.001; η^2^_p_ = 0.73; Fig. 2d). As shown in Fig. 2, it was detected an increase of AP and ML MOS, indicating a decrease in dynamic stability, during PT compared to the other two conditions (AP MOS: PT vs. BLK, *p* = 0.001, PT vs. UP, *p* = 0.001; ML MOS: PT vs. BLK, *p* = 0.002, PT vs. UP, *p* < 0.001). At the same time, a reduction of anterior displacement of the COM (AP COM) and a lateral displacement toward the swing side (ML COM, i.e. same direction of waist pulling) were more manifested during PT than in BLK and UP conditions (AP COM: PT vs. BLK, *p* = 0.001, PT vs. UP, *p* < 0.001; ML COM: PT vs. BLK, *p* < 0.001, PT vs. UP, *p* < 0.001).

### Preparatory-phase parameters

Mixed two-way ANOVA showed a main effect of condition for thrust duration (F_2,36_ = 11.686, *p* = 0.001; η^2^_p_ = 0.39), thrust amplitude (F_2,36_ = 29.293, *p* < 0.001; η^2^_p_ = 0.62), peak vertical force (F_2,36_ = 38.135, *p* < 0.001; η^2^_p_ = 0.68), peak anteroposterior force (F_2,36_ = 36.943, *p* < 0.001; η^2^_p_ = 0.67), peak mediolateral force (F_2,36_ = 24.499 *p* < 0.001; η^2^_p_ = 0.58) but not for the preparatory duration (F_2,36_ = 2.493, *p* = 0.116; η^2^_p_ = 0.12). As shown in Table 1, PT was significantly different with respect to the other two conditions for most of the preparatory-phase parameters. Significant differences were also found between BLK and UP for thrust amplitude, peak vertical and mediolateral forces (Table 1).

**Table 1:** Gait initiation parameters of each age group in all experimental conditions

		BLK	UP	PT	*F*	*p*	*η*^*2*^_*p*_
Thrust duration (ms)	Y	237 ± 39	254 ± 35	335 ± 75^a,b^	13.46	0.002	0.599
	O	264 ± 44	240 ± 18	280 ± 56	2.13	0.155	0.192
Thrust amplitude (cm)	Y	4.2 ± 1.0	5.1 ± 1.6^a^	9.2 ± 2.7^a,b^	18.80	0.001	0.676
	O	3.3 ± 1.1	4.2 ± 1.0^a^	5.4 ± 1.8^a,c^	12.47	0.002	0.581
Preparatory duration (ms)	Y	515 ± 75	501 ± 77	580 ± 141	3.07	0.103	0.254
	O	550 ± 115	471 ± 45	487 ± 73	2.92	0.105	0.245
Vertical force (BW)	Y	0.66 ± 0.04	0.71 ± 0.08	0.85 ± 0.09^a,c^	20.44	0.000	0.694
	O	0.63 ± 0.08	0.70 ± 0.09^a^	0.74 ± 0.09^a,c^	23.26	0.000	0.721
Anteroposterior force (BW)	Y	0.05 ± 0.005	0.06 ± 0.006	0.08 ± 0.005^a,b^	28.05	0.000	0.757
	O	0.05 ± 0.01	0.06 ± 0.01	0.06 ± 0.009^a^	11.77	0.001	0.567
Mediolateral force (BW)	Y	0.07 ± 0.005	0.08 ± 0.005	0.11 ± 0.01^a,b^	16.43	0.002	0.646
	O	0.05 ± 0.003‡	0.06 ± 0.004^a,c^	0.07 ± 0.008^a,c^	9.09	0.010	0.502
Step duration (ms)	Y	366 ± 41	352 ± 53	212 ± 44^a,b^	52.65	0.000	0.854
	O	362 ± 50	329 ± 57*	187 ± 60^a,b^	59.13	0.000	0.868
Step length (cm)	Y	54.4 ± 6.0	52.5 ± 7.5	43.7 ± 9.5^a^	6.64	0.010	0.424
	O	47.3 ± 7.3‡	48.6 ± 10.9	32.3 ± 10.4^a,b,c^	23.82	0.000	0.726
Step width (cm)	Y	0.5 ± 2.7	0.6 ± 2.9	− 13.0 ± 6.4^a,b^	31.66	0.000	0.779
	O	− 1.1 ± 2.4	− 0.4 ± 1.6	− 12.4 ± 3.6^a,b^	91.33	0.000	0.910
Step height (cm)	Y	6.8 ± 1.4	6.9 ± 1.6	9.6 ± 1.8^a,b^	12.05	0.002	0.573
	O	7.0 ± 1.6	7.1 ± 1.7	7.7 ± 1.6 ‡	1.94	0.180	0.177
Step velocity (m/s)	Y	1.56 ± 0.18	1.53 ± 0.20	1.99 ± 0.38^a,b^	11.88	0.004	0.569
	O	1.41 ± 0.25	1.50 ± 0.24	1.72 ± 0.40	3.97	0.071	0.306

*BLK* blocked condition, *UP* unperturbed condition, *PT* perturbed condition *Y* young, *O* older

^a^Significantly different from BLK

^b^Significantly different from UP

^c^Significantly different from Young (between-group comparison, *p* < 0.05)

Thrust duration was longer during PT than BLK by 97.4 ms (*p* = 0.014) and during PT than UP by 80.8 ms (*p* = 0.006) in young participants, while no significant changes over conditions were observed in older participants. For thrust amplitude, young participants exhibited a difference of 4.92 cm comparing PT with BLK (*p* = 0.002) and a difference of 4.02 cm between PT and UP (*p* = 0.013), while in older participants, the difference between PT and BLK was 2.13 cm (*p* = 0.008) and no significant changes were detected comparing PT and UP. Finally, there was an increase for the three components of ground reaction force from BLK to PT in both young and older participants, with a significant between-group difference for vertical and mediolateral force (Table 1).

Regarding the comparison between UP and BLK conditions, significant differences were found for thrust amplitude that was higher during UP than BLK in both young (*p* = 0.043) and older participants (*p* = 0.013), and for the vertical and mediolateral forces that were higher during UP than BLK only in older persons (*p* = 0.001 and *p* = 0.006, respectively).

### Stepping-phase parameters

Mixed two-way ANOVA showed a main effect of condition on all stepping phase parameters: step duration (F_2,36_ = 111.525, *p* < 0.001; η^2^_p_ = 0.86), step length (F_2,36_ = 25.738, *p* < 0.001; η^2^_p_ = 0.59), step width (F_2,36_ = 87.114, *p* < 0.001; η^2^_p_ = 0.83), step height (F_2,36_ = 13.677, *p* < 0.001; η^2^_p_ = 0.43) and step velocity (F_2,36_ = 14.127, *p* < 0.001; η^2^_p_ = 0.44). As shown in Table 1, PT was significantly different from each of the other two conditions for all parameters, with participants of both age groups initiating to walk with a shorter, less long lasting, faster and narrower step during PT compared to BLK and UP.

Interestingly, pairwise comparison between conditions revealed that step height was greater during PT than during BLK (gap of 2.81 cm;* p* = 0.022) and UP (gap of 2.73 cm; *p* = 0.004) in young participants, but no significant differences were detected for older participants.

### Ankle muscle activity

**Fig. 3:**
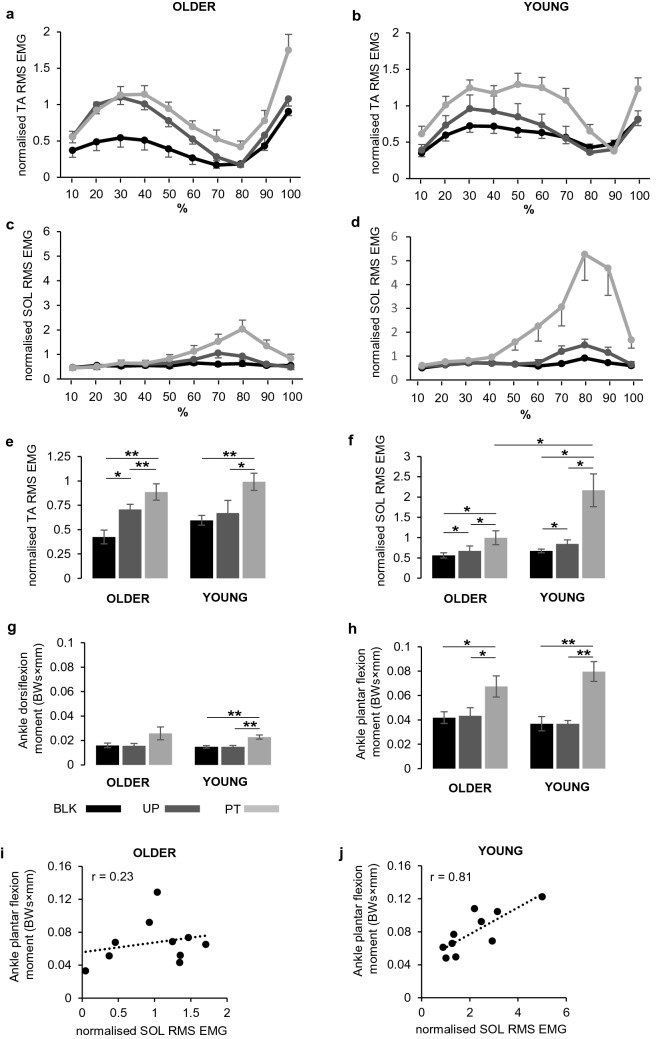
Magnitude of muscles activity computed as the root mean square (RMS) of the processed EMG signal over 10% consecutive windows, during the preparatory phase, for each condition. Plots represent the activity of tibialis anterior (TA) in older (**a**) and young (**b**) participants and the activity of soleus (SOL) in older (**c**) and young (**d**) participants. A summary of statistic results is showed in **e** for TA and in **f** for SOL. Data are reported as means and standard errors. Abbreviations, symbols and labels as in Fig. 2

Figure 3a–d shows the normalised average values of EMG-RMS amplitude for each 10% window of the preparatory phase for both young and older groups. Visual inspection of the EMG-RMS PT traces for the TA muscle (Fig. 3a,b) suggests that, in both young and older participants, its activation increased during the first half of the preparatory phase, then dropped to a minimum during the second half of the phase and finally started to increase again. It is interesting to note that older participants showed a preliminary increase of TA muscle activity in the UP condition, while young participants did not. Activation of the SOL muscle (Fig. 3c,d) remained low in the first half of the preparatory phase and then increased during the second half of the phase in both young and older participants, with a higher increase of EMG-RMS in young compared to older participants.

Figure 3e,f shows EMG data summarised as averages over the RMS windows for TA muscle (Fig. 3e) and for SOL muscle (Fig. 3f). Differences between groups and across conditions and RMS windows for the TA and for the SOL muscle are reported in Table 2. The TA muscle showed significant differences for the condition, RMS and all the interaction combinations. All main factors and interactions were significant for the SOL muscle. In this case, significant differences detected for the group depended mainly on the larger EMG-RMS amplitude showed by the young with respect to the older participants, for the PT condition (*p* = 0.016).

**Table 2:** Summary of three-way ANOVA applied on normalised EMG-RMS data

	TA			SOL		
Factors	*F*	*p*	η^2^_p_	*F*	*p*	η^2^_p_
Group	0.75	0.398	0.04	5.655	**0.029**	0.24
Condition	22.751	** < 0.001**	0.56	21.843	** < 0.001**	0.55
Condition × Group	1.375	0.266	0.07	7.134	**0.002**	0.28
RMS	28.602	** < 0.001**	0.61	20.581	** < 0.001**	0.53
RMS × Group	6.493	**0.001**	0.27	5.803	** < 0.001**	0.24
Condition × RMS	6.645	** < 0.001**	0.27	15.104	** < 0.001**	0.46
Condition × RMS × Group	2.369	**0.036**	0.12	4.964	**0.01**	0.22

Statistically significant values are reported in bold

*TA* tibialis anterior muscle, *SOL* soleus muscle, *RMS* root mean square value

*F*, effect size; *p*, significance—probability value; η^2^_p_, partial eta squared

The individual contribution of each condition in differentiating the two groups was evaluated by separated one-way ANOVAs for each muscle with multiple comparisons over the conditions, applying Bonferroni corrections. Significant main effects of condition were observed in both young and older groups for TA (older: F_2,18_ = 17.719, *p* = 0.001; η^2^_p_ = 0.66; young: F_2,18_ = 10.066, *p* = 0.003; η^2^_p_ = 0.53) and SOL muscles (older: F_2,18_ = 12.638, *p* = 0.004; η^2^_p_ = 0.58; young: F_2,18_ = 9.580, *p* = 0.011; η^2^_p_ = 0.52). For the TA muscle, all pair comparisons between conditions were significantly different for the older group (PT vs. BLK, *p* = 0.005; PT vs. UP, *p* = 0.007; BLK vs. UP, *p* = 0.012), while the young group showed significant differences when PT condition was compared with BLK (*p* = 0.004) or with UP (*p* = 0.010). For the SOL muscle, significant differences were found over all the comparisons for both older (PT vs. BLK, *p* = 0.014; PT vs. UP, *p* = 0.018; BLK vs. UP *p* = 0.039) and young (PT vs. BLK, *p* = 0.030; PT vs. UP, *p* = 0.047; BLK vs. UP *p* = 0.037) groups.

To evaluate the contribution of each EMG-RMS window to the significant changes observed between groups and over conditions, pair-wise comparisons across the 10 RMS windows were evaluated for each condition and in each group, applying Bonferroni correction. Except few cases, most significant changes occurred between the minimum and maximum value across the EMG-RMS trace (see Fig. 3a–d); therefore, we reported the statistical results obtained comparing the minimum–maximum pairs for each condition. For the TA muscle (Fig. 3a,b), the lowest value of EMG amplitude occurred in the range of EMG-RMS windows between 70 and 90% of the preparatory phase, while the maximum value occurring before the minimum comprised between 30 and 40% of the preparatory phase. There were significant differences between the lowest and highest values of the EMG-RMS for UP (*p*  < 0.001) and PT (*p* = 0.004) in older participants, and only for PT (*p* = 0.004) in young participants. An opposed pattern was detected for the SOL muscle (Fig. 3c,d), with the highest value of EMG-RMS found between 70 and 80% of the preparatory phase, and the lowest value (before the maximum) found between 10 and 20% of the preparatory phase. Significant changes between the minimum and maximum were detected only in the young group for the PT condition (*p* = 0.048).

### Ankle joint moments and their relationship with muscle activity

Figure 3g–j shows peak ankle moments in both directions of plantar flexion and dorsiflexion (Fig. 3g,h) and their relationship with muscular activity (Fig. 3i,j). A 3-way ANOVA comparing group, moment direction and condition revealed a main effect of moment with ankle plantar flexion moment being higher than ankle dorsiflexion moment (F_1,36_ = 71.618, *p* < 0.001; η^2^_p_ = 0.67). There was a main effect of condition (F_1.63,58.51_ = 45.253, *p* < 0.001; η^2^_p_ = 0.56) and a significant interaction between moment and condition (F_1.63,58.51_ = 15.244, *p* < 0.001; η^2^_p_ = 0.30). This interaction depended mainly on the important increase of plantar flexion during PT condition in both groups (Fig. 3h), with significant differences comparing PT with BLK and UP conditions in older (PT vs. BLK: p = 0.015; PT vs. UP *p* = 0.014) and in young participants (PT vs. BLK: *p* = 0.001; PT vs. UP *p* = 0.001). Similar significant differences, but with lower values, were observed for the dorsiflexion moment in young participants only (PT vs. BLK: *p* = 0.003; PT vs. UP *p* = 0.001). Finally, to quantify the relationship between muscle activity and joint moments, linear regressions were performed between the maximum EMG activity recorded in each muscle and both ankle joint moments for each group. Figure 3i, j shows that a strong correlation was found when changes in SOL EMG activity were associated with changes in ankle plantar flexion torque in the young group only (*r* = 0.81, *p* < 0.001). No significant correlations were obtained for the other comparisons.

### Correlation between postural and stepping parameters

Partial correlation coefficients between preparatory- and stepping-phase parameters for each condition and group are reported in Table 3. Step duration showed an overall good correlation with preparatory-phase parameters in both age groups during BLK, but this association was almost lost during PT. Similarly, young participants’ step length was generally correlated with timing parameters of the preparatory phase in BLK but this association was lost in PT. On the other hand, older participants’ step length was associated with preparatory-phase parameters, particularly thrust amplitude and peak force, in both BLK and PT. Step width was generally well correlated with preparatory parameters in both groups during BLK, but this association was maintained only by young and not by older participants during PT. In both groups, step height and velocity showed a strong link with most preparatory-phase parameters during PT. In young participants, ML COM position and ML MOS increased their link with preparatory-phase parameters during PT compared to BLK; on the contrary, older participants showed weak or no correlations between these two measures of stability and the preparatory-phase parameters.

**Table 3:** Partial correlation coefficients between preparatory and stepping phase parameters

		Step duration	Step length	Step width	Step height	Step velocity	AP COM	ML COM	AP MOS	ML MOS
BLK										
Y	PD	0.23*	− 0.29**	− 0.02	0.42**	− 0.32**	− 0.41**	0.37**	0.27*	− 0.39**
	TD	0.01	− 0.30**	− 0.28**	0.40**	0.05	− 0.21*	− 0.03	− 0.13	− 0.23*
	TA	− 0.22*	− 0.09	− 0.43**	0.27**	0.66**	0.18	− 0.36**	− 0.60**	− 0.17
	PVF	− 0.22*	− 0.20	− 0.37**	0.29**	0.59**	0.12	− 0.30**	− 0.62**	− 0.01
O	PD	− 0.06	− 0.17	0.37**	− 0.16	− 0.30**	0.01	0.27**	0.09	− 0.10
	TD	− 0.11	− 0.25*	− 0.01	0.17	0.04	− 0.03	0.09	− 0.24*	− 0.22*
	TA	0.14	0.23*	− 0.40**	0.30**	0.60**	0.28**	− 0.28*	− 0.51**	− 0.07
	PVF	0.01	0.29**	− 0.22	0.44**	0.75**	0.30**	− 0.08	− 0.38**	0.12
UP										
Y	PD	− 0.08	− 0.10	0.37**	0.23*	− 0.12	− 0.14	0.50**	0.11	− 0.22*
	TD	− 0.08	− 0.39**	0.07	− 0.09	− 0.34**	− 0.30**	0.19	− 0.24*	− 0.34**
	TA	0.46**	0.44**	0.24*	− 0.09	0.07	0.33**	0.25*	0.30**	0.07
	PVF	0.21*	0.41**	0.35**	− 0.19	0.21*	0.47**	0.30**	0.10	0.42**
O	PD	− 0.14	− 0.31**	0.18	0.26**	− 0.21*	− 0.19	0.32**	− 0.09	0.05
	TD	0.09	0.01	0.02	0.25*	0.15	0.02	0.21*	0.08	− 0.10
	TA	0.40**	0.44**	0.09	0.13	0.47**	0.40**	0.19	0.16	− 0.21*
	PVF	0.10	0.43**	0.22*	0.25*	0.67**	0.46**	0.31**	− 0.05	0.24*
PT										
Y	PD	− 0.27**	0.05	0.33**	0.33**	0.23*	0.42**	0.48**	− 0.35**	− 0.26**
	TD	− 0.20	0.11	− 0.00	0.46**	0.43**	0.48**	0.14	− 0.39**	− 0.26**
	TA	− 0.15	− 0.06	0.20*	0.35**	0.33**	0.25*	0.43**	− 0.46**	− 0.62**
	PVF	− 0.23*	− 0.20	0.17	0.25*	0.34**	0.31**	0.24*	− 0.66**	− 0.22**
O	PD	− 0.13	0.05	0.02	0.48**	0.39**	0.10	0.02	0.09	− 0.12
	TD	− 0.04	0.15	− 0.09	0.47**	0.42**	0.17	-0.11	0.13	− 0.16
	TA	0.20	0.36**	− 0.04	0.41**	0.40**	0.41**	-0.01	0.11	− 0.35**
	PVF	0.11	0.23*	0.04	0.36**	0.36**	0.39**	0.03	− 0.20*	− 0.02

*BLK* blocked condition, *UP* unperturbed condition, *PT* perturbed condition, *Y* young, *O* older, *PD* preparatory phase duration, *TD* thrust duration, *TA* thrust amplitude, *PVF* peak vertical force

*p < 0.05; **p < 0.01

## Discussion

In this study, we compared the ability of young and older adults to preserve stability in response to an unexpected external perturbation that was delivered within the preparatory phase of gait initiation by pulling the participants’ waist towards the stepping limb, i.e. opposite to the COM displacement towards the stance limb that typically occurs in this phase (Mouchnino et al. 2012; Mille et al. 2014). In line with the first hypothesis of the study, young participants were able to modify their response to the perturbation since the early stages of gait initiation to preserve adequate levels of whole-body stability during the first step. The second hypothesis of the study was partially met as older adults showed a compromised ability to actively respond to the perturbation in the early preparatory phase of gait initiation, which in turn affected step execution parameters but (contrary to our hypothesis) not dynamic stability at the end of the step. In detail, despite older participants responded to the perturbation with lower increases in thrust amplitude, thrust duration, vertical reaction force and SOL muscle activity with respect to young participants, they took a shorter and lower first step and reached greater dynamic stability than young in the perturbed trials, which might reflect a compensatory safety strategy attempting to recover balance by the end of the first step. Another main finding was that the perturbation-induced changes were correlated with measures of dynamic stability at the end of the first step, e.g., AP and ML MOS, in young participants, but this correlation was weaker or even lacking in the older participants. Therefore, a plausible interpretation of these findings is that young participants took more time than older to deliver an efficient biomechanical response to the perturbed posture to restore the anticipatory mechanisms disrupted by the perturbation and their link with dynamic stability at the end of the first step. On the other hand, older participants were unable to modify the timing of the preparatory phase in response to the perturbation and, hence, showed a diminished modulation of their anticipatory response, while mainly relying on compensatory mechanisms and adopting a slower, shorter and lower step in the attempt to preserve adequate levels of dynamic stability at the end of the first step.

### Biomechanical adjustments relevant to increase first-step stability following perturbation

A first aspect supporting that increased preparatory response time (e.g., thrust duration) following the perturbation may help restoring the disrupted anticipatory mechanisms and, hence, the first-step stability concerns the pattern of muscular activation observed in this study. In particular, both young and older participants showed a characteristic response pattern in the lower leg muscles following the perturbation, involving decreased TA and increased SOL muscle activity. Based on the active functional role of these muscles in controlling the COM position during locomotor activities (John et al. 2012; Cronin et al. 2013), the above pattern would tend to generate an ankle plantar flexion moment and likely act to minimize disrupting effects of the perturbation by slowing down the forward lean of the body that normally occurs in the controlled fall of gait initiation (Mann et al. 1979; Laudani et al. 2006). This schema was further supported by our results showing higher ankle plantar flexion moment compared to dorsi flexion moment in all experimental conditions, with a remarkable increase during perturbed trials for both groups.

Older participants in the study showed a lower increase of SOL muscle activity following the perturbation compared to young, with the ankle plantar flexion moment following a similar trend despite the between-group difference was not significant. Noteworthy, the regression analysis revealed that changes in SOL activity during PT in young participants showed a strong correlation (*r* = 0.81; *p* < 0.001) with changes in ankle plantar flexion moment, while no significant correlation was found for older participants. This suggests that the ankle plantar flexion moment observed in young participants would depend mainly on the contribution of the SOL activity, while the moment produced by older participants could include a strong contribution from forces originated by non-muscular tissues, such as elastic and non-elastic joint tissue, that increase with ageing (Rasske and Franz 2018; Hirata et al. 2020). Overall, our results show that the SOL activity and the consequent modulation of ankle plantar flexion plays a critical role in differentiating the response to perturbation between young and older people.

This is particularly relevant considering the functional key role played by the SOL in walking (Cronin et al. 2013) and by plantar flexors in controlling mediolateral ground reaction force during the latter half of stance over a wide range of speeds (John et al. 2012); hence, an age-related reduction in the response of SOL muscle activity following the perturbation is likely to pose older individuals at greater risk of imbalance just before initiating the step. This is in line with previous studies showing a diminished lower limb muscle activity during the preparatory phase of unperturbed gait initiation in older compared to young adults (Polcyn et al. 1998; Mickelborough et al. 2004). An age-related deterioration in lower limb muscles activation in response to postural perturbations has been previously reported in older persons during other balance and locomotor tasks and it is considered as a major mechanism affecting the risk of falling in the elderly (see review by Potocanac and Duysens 2017). Noteworthy, and in agreement with the current study results, older individuals showed impaired activity of ankle plantar flexor muscles in controlling the COM acceleration experienced throughout balance recovery from forward loss of balance by stepping (Graham et al. 2017).

Another aspect concerns the differences between-age groups in the spatial characteristics of the first step. The shorter and lower step showed by older participants following the perturbation is in line with findings from previous studies focusing on reactive stepping following mediolateral perturbations during standing and walking in place (Maki et al. 2000a, b), steady-state walking (McIntosh et al. 2017) and gait initiation (Shulman et al. 2018). As also suggested in these studies, these changes in step characteristics may reflect the need for a more cautious stepping strategy to reduce the risk of losing balance and falling (Winter et al. 1995). As the lack of relevant changes in anticipatory adjustments might have increased the level of balance challenge and decreased stability during the preparatory phase in older participants, hence, they were constrained to react and modify the first-step parameters to preserve dynamic stability at the end of the first step.

### Role of anticipatory and compensatory adjustments in response to external perturbation

From a motor control perspective, the external perturbation disrupted the typical anticipatory adjustments aimed to stabilize the first step, with the consequence that the control of balance during the leg movement could be based on online compensatory reactions. Alternatively, participants could implement an internal representation of the external perturbation and produce an update of anticipatory adjustments that contemplates the possible occurrence of the external disturbance. Indeed, the current work and the relevant literature provide several points indicating that older participants adopted a compensatory strategy, while young participants tried to engage a predictive implementation. The fact that lateral thrust duration was prolonged and thrust amplitude increased following delivery of the perturbation in young participants suggests that the CNS estimated the potential destabilising effects of perturbed anticipatory adjustments to modify postural events upon locomotor stepping execution and ensuring stability of the planned movement. This is in line with the results and the conclusions of the study by Mille et al. (2014) and Mouchinino et al. (2012), which found that both thrust duration and amplitude were affected by the unexpected perturbation in young participants, with similar changes to those observed in the current study. According to an interactive model of posture and locomotion coordination (Massion 1992; Schepens and Drew 2003), Mille et al. (2014) suggested that these results are consistent with a role of motor prediction in posture and locomotion coupling, which involves an internal model of the interactions between the one’s own body and the external world (Wolpert et al. 1995; Kawato 1999). In our study, this schema is supported by the good correlation between preparatory-phase parameters and measures of stability of the first step observed in young people during perturbed trials. On the other hand, this association was weak or absent in older participants who initiated the first step (i.e. toe-off of the stepping leg) motor program with no delay and irrespective of any potential destabilising effects of perturbed APAs. This behaviour indicated that no prediction in posture and locomotion coupling were engaged by older subjects and the response to perturbation was likely to rely mainly on compensatory reactions. Previous studies have suggested that older adults adopt APAs to ensure stability in performing demanding tasks, such as change direction during walking (Paquette et al. 2008; Mangano et al. 2020) or following postural perturbations (Kanekar and Aruin 2014), with a marginal age-related decline. However, older adults have shown a diminished ability in the use and efficacy of APAs in ensuring stability following external perturbations in the frontal plane (Claudino et al. 2013). Therefore, the findings of the present study suggest that older adults may have difficulties in utilising APAs due to a lack of the necessary coupling between APAs and the main focal movement of stepping. This may put additional demands to the postural control system of older individuals by forcing them to respond to the perturbation through compensatory rather than anticipatory movements (Kanekar and Aruin 2014).

The differences in response strategies implemented by young participants compared to older emerge also from the modulation in TA muscle activity observed in the two groups. The EMG activity of the stepping leg TA muscle was increased during the unperturbed trials compared to the blocked gait initiation trials in older participants to a greater extent than in young participants. Thus, when older individuals were waiting for the perturbation, they increased the level of muscle activation regardless of the occurrence of the perturbation. On the same line, the enhanced contribution of SOL activity in generating ankle plantar flexion moment in young during perturbed trial with respect to older, may improve the individual chance/ability of modulating anticipatory responses (Le Mouel and Brette 2019). This is in line with previous studies showing that fear of falling can alter postural control parameters and increase muscle activation levels around the ankle during quiet stance and gait (Yiou et al. 2011; Young and Mark Williams 2015). Reasonably, such stiffening strategies can be interpreted as a generic compensatory response aimed at overcoming potential posture destabilization. As argued by Young and Williams (2015), however, an unspecific stiffening strategy may reduce the capacity to achieve dynamic activities of daily life where humans are required to interact with features of the environment in a complex and flexible manner; in turn, this might increase the likelihood of misguiding balance and failing to produce a sufficient response to external perturbations, as it was the case in the present study.

### Strengths and limitations

A strength of the study was the strict inclusion criteria for older participants that were classified “medically stable” for exercise studies as proposed by Greig et al. (1994) and at “low fall risk” based on the Berg Balance Scale to assess both static and dynamic balance skills (Berg et al. 1989). For the first time, hence, this study uncovered and reported the neuro-mechanical postural strategies adopted by low-risk older individuals to preserve dynamic stability despite diminished ability to respond in an active and timely manner to unexpected real-body perturbation delivered within the early stages of gait initiation. Specifically, the robust experimental setup adopted in the present study allowed highlighting the population/sample specific deficits in the ability to modify the pre-planned anticipatory postural adjustments, while dynamic stability was nevertheless preserved likely by adjusting the step characteristics. These results, hence, should be taken into account when designing appropriate training and rehabilitation programs to selectively target the preparation of movement during transitory locomotor activities, such as gait initiation, in low fall risk individuals to prevent their development into medium or high risk of falling. On the other hand, a limitation of the present study was that sample size (*n*: 20) approached the minimum necessary to obtain a significant effect size based on a priori analysis. A higher number of participants would have ensured to reduce the variability of data and perhaps uncover more subtle age-induced effects.

## Conclusion

The results of this study indicated that responses to unexpected perturbation occurring in the preparatory phase of the gait initiation are age-dependent. Young individuals delayed the timing of their anticipatory adjustments and modulated the responses to preserve balance of the upcoming step, while older individuals showed a compensatory reaction to the perturbation, with their responses having weak or no association with the first-step stability. It is possible that the older group behaviour depends on an age-related deterioration of APAs, but we cannot exclude that older people need more practice to incorporate the forthcoming external perturbation and produce predictive responses. Further studies with an experimental learning-based design could clarify this issue.

## Abbreviations

APA: Anticipatory postural adjustment
COP: Centre of pressure
COM: Centre of mass
GRF: Ground reaction force
EMG: Electromyography
SOL: Soleus muscle
TA: Tibialis anterior muscle
RMS: Root mean square
MOS: Margin of stability
ANOVA: Analysis of variance
